# Development of CMX001 for the Treatment of Poxvirus Infections

**DOI:** 10.3390/v2122740

**Published:** 2010-12-17

**Authors:** Randall Lanier, Lawrence Trost, Tim Tippin, Bernhard Lampert, Alice Robertson, Scott Foster, Michelle Rose, Wendy Painter, Rose O’Mahony, Merrick Almond, George Painter

**Affiliations:** Chimerix, Inc., 2505 Meridian Parkway, Suite 340, Durham, North Carolina, NC 27713, USA; E-Mails: ltrost@chimerix.com (L.T.); ttippin@chimerix.com (T.T.); blampert@chimerix.com (B.L.); arobertson@chimerix.com (A.R.); sfoster@chimerix.com (S.F.); mrose@chimerix.com (M.R.); wpainter@chimerix.com (W.P.); romahony@chimerix.com (R.O.); malmond@chimerix.com (M.A.); gpainter@chimerix.com (G.P.)

**Keywords:** CMX001, smallpox, dsDNA virus, antiviral, cidofovir, variola

## Abstract

CMX001 (phosphonic acid, [[(S)-2-(4-amino-2-oxo-1(2H)-pyrimidinyl)-1-(hydroxymethyl)ethoxy]methyl]mono[3-(hexadecyloxy)propyl] ester) is a lipid conjugate of the acyclic nucleotide phosphonate, cidofovir (CDV). CMX001 is currently in Phase II clinical trials for the prophylaxis of human cytomegalovirus infection and under development using the Animal Rule for smallpox infection. It has proven effective in reduction of morbidity and mortality in animal models of human smallpox, even after the onset of lesions and other clinical signs of disease. CMX001 and CDV are active against all five families of double-stranded DNA (dsDNA) viruses that cause human morbidity and mortality, including orthopoxviruses such as variola virus, the cause of human smallpox. However, the clinical utility of CDV is limited by the requirement for intravenous dosing and a high incidence of acute kidney toxicity. The risk of nephrotoxicity necessitates pre-hydration and probenecid administration in a health care facility, further complicating high volume CDV use in an emergency situation. Compared with CDV, CMX001 has a number of advantages for treatment of smallpox in an emergency including greater potency *in vitro* against all dsDNA viruses that cause human disease, a high genetic barrier to resistance, convenient oral administration as a tablet or liquid, and no evidence to date of nephrotoxicity in either animals or humans. The apparent lack of nephrotoxicity observed with CMX001 *in vivo* is because it is not a substrate for the human organic anion transporters that actively secrete CDV into kidney cells. The ability to test the safety and efficacy of CMX001 in patients with life-threatening dsDNA virus infections which share many basic traits with variola is a major advantage in the development of this antiviral for a smallpox indication.

## 1. Introduction

Variola virus, the etiologic agent of smallpox, is a CDC Category A pathogen. Smallpox is one of the most pestilent diseases feared by mankind since antiquity, and historically has been associated with the periodic occurrence of widespread, often devastating epidemics. The fatality rate associated with the disease was reported to be roughly 30%, and a significant number of survivors were disfigured and/or blinded. However, historical data were derived from a population in which the disease was endemic. The last naturally acquired case of smallpox occurred in 1977 and the last laboratory acquired infection in 1978 [1]. In 1980, the World Health Organization (WHO) reported that, due to their aggressive worldwide vaccination program, smallpox had been eradicated [2]. Subsequent to this declaration, the worldwide vaccination campaign was stopped (the last vaccinations of the general population in the United States were in 1972 and in the rest of the world in 1984) because of side effects associated with smallpox vaccine [2]. As a result, herd immunity has been lost in the ensuing 30+ years, leaving most of the world’s population highly vulnerable to morbidity and mortality from variola infection.

In the wake of the terrorist attacks on September 11, 2001, and the anthrax attacks using the U.S. Postal Service to deliver letters containing *Bacillus anthracis* in October of 2001, the United States began in earnest to consider the impact of reintroduction of smallpox via a deliberate release into a highly susceptible population. Within weeks of September 11, 2001, the U.S. government decided to purchase adequate smallpox vaccine, 300 million doses, to immunize the entire population of the United States. If the public health infrastructure is prepared for the release of smallpox into the general population, many people could indeed be protected by vaccination. However, if vaccination is delayed by as few as three to four days after exposure, there may be little benefit. As in the case of mortality figures, the impact of delay on the development of a protective immune response after vaccination is based on observations made during the WHO eradication campaign under natural transmission conditions in a population in which the disease was endemic [3]. The impact of infection with a large inoculum of variola virus on the window of opportunity for vaccinating primary cases is unknown in the current highly susceptible population. In any event, many secondary cases of smallpox will likely occur and the disease could become widespread before primary cases of smallpox are identified, in part due to the lack of familiarity of disease signs and symptoms among the current generation of healthcare professionals. Diagnosis for public health response purposes will most likely be made after the onset of lesional disease. Consequently there is a need for antiviral drugs to treat patients who are diagnosed with smallpox after the appearance of lesions. Ultimately the same drugs will be used prophylactically/preemptively in exposed but asymptomatic populations to delay the progression of infection and increase the window of opportunity for vaccination and the development of protective immunity.

In response to the threat of variola release, the Interagency Working Group on smallpox, a U.S. government group made up of officials at the Assistant Secretary level, recommended in 1998 that two antiviral drugs for treating smallpox with different mechanisms of action be developed. At least one of these drugs was to be orally available [4]. However, in addition to the indication for the treatment of smallpox in the general population, the drug would be the first line of defense for the tens of millions of people who are at risk of life-threatening vaccinia infections if vaccinated with a live attenuated virus vaccine such as the currently stockpiled ACAM2000. These populations include pregnant women and many groups of patients whose immune systems are weakened (e.g., AIDS patients, organ transplant recipients and patients on cancer therapy). Another possible threat is suggested by the demonstration that genetically engineered interleukin (IL)-4-positive strains of mousepox virus are lethal in mice, despite prior vaccination [5]. This modification, if applied to variola virus, could produce a strain that causes severe illness or death even in the vaccinated population. It is clear that the complete defense of the country against a terrorist attack using variola virus, whether modified or not, requires safe, easily administered antiviral drugs ready for use in the Strategic National Stockpile.

## 2. The Ideal Product Profile for Use in a Public Health Emergency

The Department of Health and Human Services through the Biomedical Advanced Research and Development Authority (BARDA) issued a request for proposals in March of 2009 (RFP-BARDA-09-35) to provide a medical countermeasure that could specifically treat symptomatic individuals exposed to smallpox. Beyond stating that the product acquired under this contract must be able to “support a public health emergency”, no specific properties, performance criteria or therapeutic profile in humans were specified. In the absence of specific guidance on product profile we identified the properties listed below as being essential for a drug to be highly effective in a public health emergency.

### 

#### Potency

Clinically, potency is best defined as the relationship between the concentration of drug achieved in the plasma and the intensity of the therapeutic effect of the drug [6]. In the case of a medical countermeasure to treat an acute viral infection in a public health emergency, the potency needs to be such that at a level of drug that is easily and reproducibly achieved in the plasma of sick patients, the antiviral effect is adequate to quickly reduce viral burden thereby reducing the morbidity of the infection and, owing to reduced viral shedding, limiting infectivity.

#### Schedule

The course of therapy necessary to suppress the virus long enough for the host adaptive immune response to develop should be as short as possible and the dosing regimen should be simple and easy to follow to ensure compliance in a public health emergency.

#### Risk of Resistance

Emergence of a resistant virus post attack may allow the unchecked spread of disease through the population. The probability that a rapidly replicating virus circulating through a highly susceptible population during an epidemic would develop resistance is high. Resistance has developed to every widely used antiviral drug to date and has had a profound impact on the development of new antiviral agents [7]. The best approach to limiting viral breakthrough has been to develop potent drugs that target key replication proteins (enzymes) with a high genetic barrier to resistance. In addition, the resistant virus that ultimately emerges from such targeted drugs may be non-virulent, especially if mutations in the active site of a critical enzyme are required for the development of clinical resistance.

#### Drug-Drug Interaction

Antiviral therapy during a smallpox attack would be used for the treatment/prophylaxis of: (1) immunosuppressed patients who were exposed and are symptomatic with smallpox and, (2) those who received smallpox vaccination, developed complications from the vaccine and consequently need antiviral therapy. These groups of people will typically require significant medical management and be on multiple medications. The ideal smallpox antiviral will need to have minimal potential for drug interactions and will not require dosage adjustment in patients taking multiple concomitant medications.

#### Formulation Characteristics

The manufacturing process for the drug needs to be robust with the potential to accommodate surge capacity if necessary. The dose formulation should be easy to produce, and stable for long-term storage under a range of conditions.

These properties represent the context in which we are developing CMX001 as a potential medical countermeasure for use in a smallpox attack.

## 3. CMX001 *in vitro* Efficacy and Resistance Testing

CMX001 is active *in vitro* against a broad range of viruses from all five families of dsDNA viruses infecting humans, including multiple species of *Orthopoxviridae*. As shown in Table 1, conjugation of the lipid to the phosphonate moiety of CDV to produce CMX001 results in significant decreases in apparent EC_50_ values relative to those observed for CDV. In the case of the orthopoxviruses, the enhancement in activity (determined as the ratio of the EC_50_ CDV/EC_50_ CMX001) ranges from a high of 271-fold for variola virus to a low of 24-fold for ectromelia virus. The increased activity of CMX001 relative to CDV is attributable to the more efficient cellular uptake of CMX001 facilitated by the lipid chain [8]. CDV is transported into cells by a relatively inefficient method, fluid phase endocytosis [9].

**Figure 1:**
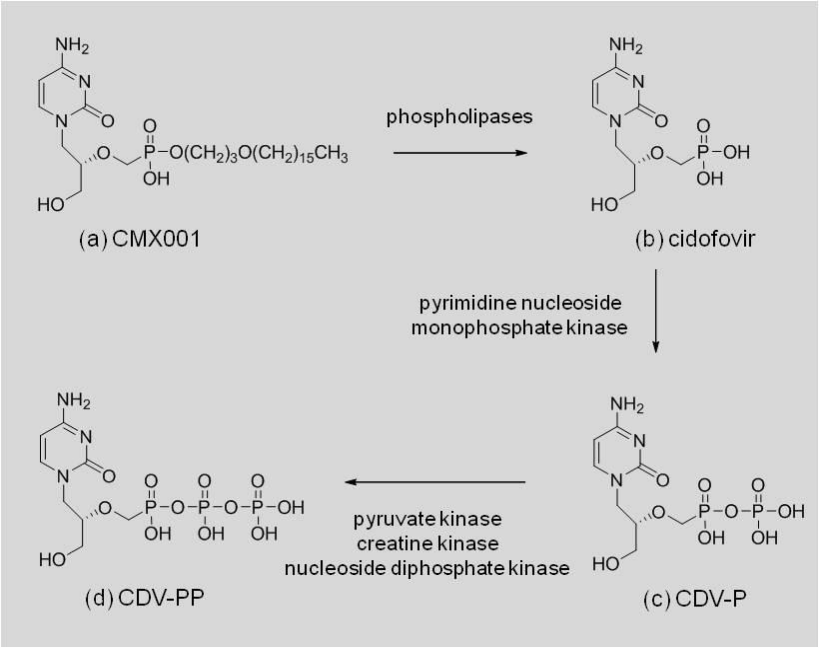
CMX001 cleavage and anabolism by host cell enzymes.

CMX001 (Figure 1a) is formed by conjugating a lipid, 3-hexadecyloxy-1-propanol, to the phosphonate moiety of CDV. Once CMX001 is inside cells, CDV (Figure 1b) is liberated by phospholipase cleavage of the lipid ester linkage and activated by two successive phosphorylations, first to cidofovir monophosphate (CDV-P, Figure 1c) and then to cidofovir diphosphate (CDV-PP, Figure 1d) [10]. The CDV-PP acts as a competitive, alternative substrate inhibitor of the DNA directed DNA polymerases encoded by the herpesvirus, adenovirus and orthopoxvirus families of double-stranded DNA viruses (dsDNA) [11–14].

The broad spectrum activity of CMX001 against various species of orthopoxviruses was anticipated based on the mechanism of action of the drug, inhibition of the virally encoded polymerase by CDV-PP, and the high level of homology for this enzyme seen within the family. The amino acid sequences of the catalytic subunit of the polymerase have been aligned for cowpox virus (CPXV, strain Brighton Red), ECTV (strain Moscow), MPXV, (strain Zaire 1979-005), VACV (strains 3737 and Western Reserve), RPXV (strain Utrecht) and VARV (strains Bangladesh 1975 and India 7129). All of the subunits are 1005 residues in length, completely overlap, and have sequence identity ranging from 98.2% to 99.1%. Sequences were obtained from Poxvirus Bioinformatics Resource Center [15] and aligned with the NCBI Protein Blast program, BLASTP 2.2.24, [16]. This level of similarity in the target enzyme also supports the use of surrogates for VARV in the animal model studies required under the Animal Rule (21 CFR 314.600) to establish the efficacy of CMX001, or more specifically the efficacy of CDV-PP, the active antiviral formed from CMX001 (Figure 1).

*In vitro* experiments to generate orthopoxvirus strains resistant to CDV have been conducted and cross-resistance to CMX001 examined. Serial passage studies of camelpox, CPXV, MPXV and VACV viruses with CDV have all generated resistant strains [26]. The extent of viral resistance is reported to be on the same order as that observed for CDV-resistant forms of herpes simplex virus and CMV (10 to 27-fold). Resistant strains of VACV (strain WR) generated after 20 to 30 passages in the presence of increasing concentrations of CDV have been shown to be cross resistant to CMX001 [27]. Mutations conferring this resistance are located in the virally encoded DNA polymerase. Additionally, the development of resistance to CDV and CMX001 in VACV leads to a significant attenuation of virulence in mice [28], suggesting that any mutants that might become dominant from drug pressure during treatment of smallpox would be attenuated and less virulent.

Twelve mutations in the polymerase domain of VACV have been associated with phenotypic resistance to CDV as shown in Table 2. The specific roles of these mutations in CDV resistance have not been uniformly elucidated and some appear to be secondary; however, 11/12 of the sites involved are completely conserved among representative members of the orthopoxviruses suggesting the data obtained in VACV can be extrapolated to other members of this family, including VARV. This observation is in accord with the overall high level of sequence homology observed across the orthopoxviruses.

## 4. Establishing Activity under the Animal Efficacy Rule

Because smallpox has been eradicated, the effectiveness of anti-VARV agents cannot be demonstrated in human clinical trials. In order to provide a development path for candidate smallpox therapeutics, the FDA promulgated the “Animal Efficacy Rule” in June of 2002 [31]. This rule establishes conditions under which animal models can be used to provide evidence of the effectiveness of an antiviral agent to treat a disease or condition for which clinical studies are impossible or unethical.

As described in 21 CFR 314, subpart I, FDA will accept data from studies in animal models as evidence of efficacy when the following criteria are met: “(1) there is a reasonably well-understood pathophysiological mechanism of the toxicity of the substance and its prevention or substantial reduction by the product; (2) the effect is demonstrated in more than one animal species expected to react with a response predictive for humans, unless the effect is demonstrated in a single animal species that represents a sufficiently well-characterized animal model for predicting the response in humans; (3) the animal study endpoint is clearly related to the desired benefit in humans, generally the enhancement of survival or prevention of major morbidity; (4) the data or information on the pharmacokinetics and pharmacodynamics of the product or other relevant data or information, in animals and humans, allows selection of an effective dose in humans.” Each of these points is discussed below in the context of the program to develop CMX001 under the Animal Efficacy Rule.

### 4.1. Pathophysiological Mechanisms

Although our understanding of the pathogenesis of variola virus infection in humans is limited, it is clear that viral replication plays a key role in the lethality of the infection. Historically, the mortality rate from ordinary-type smallpox was approximately 30% (5–50%). Mortality correlated with the density of pock lesions on the skin, which almost certainly correlated with the intensity of the secondary viremia. Thus, an intervention that reduces viral replication should also reduce lesion count and increase survival. The antiviral mechanism by which CMX001 prevents or substantially reduces the toxicity of orthopoxviruses is understood. The drug arrests disease progression by blocking viral DNA replication and thereby reducing viral burden [13,30,32].

### 4.2. Efficacy in Animal Models

The optimal model for a human virus infection is an animal that can be naturally infected with the same virus; however, humans are the only natural host for VARV. Since the target enzyme of CMX001 is highly conserved, orthopoxviruses that naturally infect the model species are the next best choice. These natural infections have several hallmarks that may make them more desirable than laboratory contrived infections, including low virus dose infections that result in high mortality, relevant routes to induce severe disease in immunocompetent hosts, and virus-encoded host response modifier genes matched to host processes. Epidemiological data suggest that VARV is most often transmitted in large respiratory droplets making respiratory transmission the most relevant [2]. There is also considerable evidence suggesting that VARV infection of humans is initiated with a small dose of virus [33]. Thus for a model, a low inoculum delivered via the upper respiratory tract is the preferred route of infection. Unfortunately, no single animal model meets these criteria and fully reproduces the clinical and pathogenic course of VARV infection in humans.

Since there is no single model of orthopoxvirus infection that completely replicates the clinical and pathophysiological course of human smallpox, CMX001 has been tested extensively in mouse, rabbit, and non-human primate models of orthopoxvirus infection. The results of these studies are summarized below.

#### CMX001 in mouse models of orthopoxvirus infection

The *in vivo* anti-orthopoxvirus activity of CMX001 has been extensively characterized in mice infected with ECTV, CPXV and VACV viruses. Ectromelia virus (mousepox) infection of mice has been used extensively as a model for orthopoxvirus pathogenesis and as a model for testing the efficacy of anti-orthopoxvirus therapeutics [34]. ECTV is a natural pathogen of mice and depending upon the mouse strain and route of inoculation, lethal infection can be initiated with a very small inoculum. Additionally, infection of mice with CPXV and VACV can be used to assess the efficacy of an antiviral against orthopoxviruses that can infect humans. The availability of an animal model(s) to help establish an effective dose for the treatment of vaccinia virus is critical in determining the potential utility of a drug in treating the side effects associated with a vaccinia derived, live attenuated virus smallpox vaccine.

A large number of CMX001 regimens have been studied in ECTV-infected A/NCR mice. In this model, a 2.5 mg/kg dose of CMX001 given once daily for five days starting 4 h post-infection provides complete protection against a lethal intranasal challenge. When treatment is started as late as five days post-infection (3–4 days prior to the death of untreated controls), mice can be completely protected from mortality with a loading dose of 10 mg/kg followed by a maintenance dose of 2.5 mg/kg every other day for 14 days. A single 20 mg/kg oral dose of CMX001 given as late as four days post-infection provided 100% protection against lethal ECTV infection [35,36].

Against CPXV infection, oral administration of 10 mg/kg of CMX001 daily for five consecutive days was 100% protective in mice infected by either the aerosol inhalation or intranasal route when dosing was initiated 4 h post-infection [22]. A single oral dose of 12.5 mg/kg was 80% protective when given as early as five days before exposure, 100% effective when given one or three days before exposure, 100% effective when given one day post–exposure, and 87% protective when given as late as three days post-exposure [37]. CMX001 therapy showed significant protection (86%) against lethal VACV infection when initiated as late as 24 h post-infection (mean day of death for vehicle controls was 6.8) [37].

#### CMX001 in a rabbitpox model

Rabbitpox virus is a subspecies of VACV that is naturally highly pathogenic in rabbits. Like other orthopoxvirus infections in their natural hosts, RPXV infection of rabbits shares many characteristics of smallpox. RPXV infection is readily transmitted between rabbits by the respiratory route and follows a pathophysiological progression similar to that of smallpox. Rabbits infected with RPXV undergo an incubation phase followed by the onset of fever, lesions, and ultimately respiratory distress and typically death [38]. Intradermal infection of rabbits allows for a controlled inoculum to be delivered while maintaining a course of disease nearly identical to that of the natural respiratory infection. Additionally, the lethal inoculum for intradermal infection is much lower than that required for intranasal infection, more closely mimicking the low inoculum required for human smallpox infection. Finally, intradermal infection results in a reliable occurrence of lesions, whereas with respiratory infection, rabbits sometimes die before rash development [39]. Ear lesions in the rabbitpox model, a result of systemic viral spread from the intradermal inoculation site on the thigh, typically appear beginning from three to five days post infection. As the desired trigger to initiate therapy for the treatment of smallpox is the onset of lesions, the reliable occurrence of lesions is a distinct advantage.

CMX001 has been studied in New Zealand White rabbits infected intradermally with a lethal inoculum of RPXV [38,40,41]. Initial studies established the efficacy of CMX001 for pre-exposure and post-exposure prophylaxis. For example, 100% of animals administered a dose of 10 mg CMX001/kg/day for five days survived when treatment was initiated one day before challenge compared to 0% survival in animals administered placebo [38]. Delayed treatment experiments showed that CMX001 can be given late in the infection cycle, after appearance of clinical signs of disease (fever and lesions), and still prevent mortality. In a representative experiment, 100% of rabbits administered a dose of 10 mg CMX001/kg/day for five days survived when treatment was initiated four days after infection compared with 0% of placebo treated animals. Treatment initiated at five days post infection also provided a survival benefit (75% of CMX001-treated compared with 0% of placebo treated).

In a series of randomized, blinded, placebo-controlled studies, administration of 20 mg/kg CMX001 initiated at the onset of lesions in the ears (mean 3.8 days post infection, range three to five days) provided a survival benefit over placebo-treated animals [40]. The results of these studies are summarized in Table 3. Even a single dose of 20 mg/kg CMX001 given at the onset of lesions provided a statistically significant survival benefit compared to placebo [40]. Note that based on AUC_0–∞_, the 20 mg/kg dose in rabbits equates to a 0.67 mg/kg dose in humans (see Table 4). Doses in this range have been used to successfully treat fulminate disseminated dsDNA infections in patients.

#### Monkeypox in Cynomolgus Monkeys

Infection of cynomolgus monkeys with MPXV produces an exanthematous disease that can be used to evaluate the effect of a drug on skin lesions, viremia, and mortality. Because this model requires a large intravenous (i.v.) dose of virus (1–5 × 10^7^ PFU of MPXV) to achieve a severe infection [42–45], the model may not reflect the pathophysiology of human disease, where the infectious dose is thought to be on the order of 100 PFU [46]. Animals manifest the first signs of illness within two to five days after infection and die within nine to 17 days (compared with six to 15 days and 16 to 22 days, respectively, in human smallpox) and have a near 100% mortality rate (as compared to 30% in humans). The distribution and progression of skin lesions in monkeys is similar to that in human smallpox. Although there may be a potential advantage to using a non-human primate as a model for a human disease, this advantage is offset by the very large intravenous inoculum required to induce severe disease.

**Figure 2:**
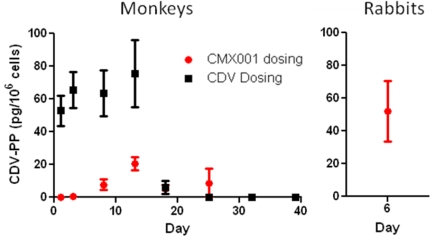
Concentrations of CDV-PP in PBMCs of healthy uninfected monkeys after i.v. administration of 20 mg/kg CDV or i.m. administration of 4 mg/kg CMX001, and in PBMCs of rabbits after oral administration of 20 mg/kg CMX001. Dosing occurred on Days 1, 6, and 11 for monkeys and on Days 1, 3, and 5 for rabbits. For averaging purposes, samples with undetectable CDV-PP were assigned a value of zero. Samples that were below the limit of quantitation (BLQ), but had detectable CDV-PP, were included in the averages at the determined value. All samples from CMX001 dosed monkeys were BLQ. Error bars represent SEM. Monkey data N = 10–12; Rabbit data N = 3.

CMX001 was tested in the i.v. inoculation MPXV model at oral doses of 2.5, 5, and 10 mg/kg administered on days 1, 3, 6, 9, and 12 post–infection. Although these doses did not reduce mortality, an explanation was provided by pharmacokinetic analysis which showed that systemic exposure of cynomolgus monkeys to CMX001 following oral administration was very low compared to that seen in other species including mice, rabbits, and humans, with humans having by far the best systemic exposure (AUC) after oral administration (see Table 4). A subsequent study of MPXV in monkeys using intramuscular (i.m.) administration of CMX001 showed an equivocal effect of treatment, despite this route resulting in a higher systemic exposure to CMX001. This result was somewhat unexpected given that i.v. CDV had been shown to be effective in the model and that both CDV and CMX001 produce the same active antiviral metabolite, CDV-PP. However, comparison of the levels of CDV-PP after i.v. CDV treatment *versus* i.m. CMX001 treatment (in healthy monkeys) revealed that the concentration of CDV-PP was much higher in the peripheral blood mononuclear cells (PBMCs) of animals treated with i.v. CDV compared to those treated with i.m. CMX001 (Figure 2). This result is consistent with intracellular conversion of CMX001 to CDV being inefficient in monkeys. Because of this metabolic difference between monkeys and humans, data obtained with CMX001 in monkeys cannot be used to model human exposure. To circumvent this issue, CDV treatment of monkeys will be used to model the exposure to the active antiviral metabolite, CDV-PP, which is common to both CDV and CMX001. This approach to using monkey model data to help establish a human dose is described under “Selection of an Effective Dose in Humans” below.

### 4.3. Selection of Endpoints

With the exception of studies of VARV in monkeys where statistically powered mortality endpoints may not be feasible, the primary endpoint in animal efficacy studies is survival to 42 days post infection. This endpoint is clearly relevant to the desired benefit in humans. In VARV studies, due to the partial mortality in the model, the ethical concerns of using large numbers of monkeys to achieve statistical significance, and the practical constraints of handling large numbers of monkeys under biosafety level 4 conditions, the proposed primary endpoint is a reduction in the number of lesions. Historically, the mortality rate from ordinary-type smallpox was positively associated with the density of pock lesions, which almost certainly correlated with the intensity of the secondary viremia. Thus, an intervention that reduces viral replication should also reduce the number of lesions and increase survival.

### 4.4. Selection of an Effective Dose in Humans

Straightforward use of different animal models of human smallpox for establishing the dose of a drug requires relatively consistent pharmacokinetic/pharmacodynamic profile of the drug across the animal model species. The pharmacokinetics (PK) of CMX001 have been determined in humans and in all animal species in which the drug has been used to study orthopoxvirus infection to date. Exposure to CMX001 after oral administration differs dramatically across species, with the lowest exposure occurring in monkeys and highest exposure occurring in humans (see Table 4). Even after achieving high plasma concentrations of CMX001 in monkeys by administration via non-oral routes, the conversion to the active moiety CDV-PP is low. The use of CDV as a surrogate for CMX001 in the monkey models avoids the differential PK and metabolism issues. As CDV-PP is the active antiviral metabolite that is common to both CMX001 and CDV, use of interspecies scaling through CDV-PP to extrapolate data from monkeys to humans is planned.

In preliminary scaling studies, treatment with three doses of 20 mg/kg CMX001 has been shown to be effective in providing a survival benefit to rabbits infected with RPXV. In healthy rabbits, this treatment regimen resulted in an average CDV-PP concentration in PBMCs of 52.1 pg/10^6^ cells when measured 24 hours after the third dose (Figure 2). Similarly, treatment with three doses of 20 mg/kg CDV has also shown efficacy in providing a survival benefit to monkeys infected with MPXV [44]. In healthy monkeys, this treatment regimen resulted in average CDV-PP concentrations in PBMCs in the range of 52.9 to 75.6 pg/10^6^ cells when measured from 12 hours after the first dose to 48 hours after the third dose (Figure 2). Based upon these results, the target for an effective intracellular concentration of CDV-PP is in the range of approximately 50 pg/10^6^ cells as measured in PBMCs.

Reliable CDV-PP concentration data in PBMCs of humans treated with CMX001 is currently not available for comparison; however, *in vitro* studies with human PBMCs indicate that these CDV-PP levels will be achieved with doses of CMX001 currently being studied in clinical trials for treatment of other dsDNA virus infections. For example, a dose of 2 mg/kg CMX001 in humans produced a C_max_ of approximately 0.6 μM (350 ng/mL) and an AUC of approximately 2650 h·ng/mL CMX001 in plasma (CMX001-102). Isolated human PBMCs incubated with 0.1 μM CMX001 for 24 hours (estimated AUC = 1348 h·ng/mL) produced a concentration of CDV-PP of approximately 79 pg/10^6^ cells at 24 hours which is within the expected efficacious range as estimated from rabbits and monkeys. Furthermore, the long half-life of CDV-PP observed in human PBMCs *in vitro* indicates that infrequent dosing with CMX001 is sufficient to maintain an effective intracellular CDV-PP concentration. Additional studies measuring CDV-PP in the PBMCs of healthy and infected animals as well as in humans are planned.

## 5. Toxicology of CMX001

Twenty-six toxicology studies have been conducted to describe the toxicological profile and secondary pharmacology of CMX001 to support its clinical development. Most studies employed dose administration by the oral route; however, CMX001 has also been administered to animals topically and by the intravenous, subcutaneous and intramuscular routes. Thirteen week studies in rats and cynomolgus monkeys have been completed and a 39-week study in monkeys is ongoing. Dose administration was daily in studies up to 14 days duration and twice weekly in the 13 and 39 week studies because it appeared that a longer dosing interval would provide relief from the dose-limiting gastrointestinal (GI) toxicity allowing administration of higher doses. Topical administration resulted in quantifiable systemic exposure and parenteral administration was not well tolerated due to injection site reactions and poor hemocompatibility.

The dose-limiting toxicity of CMX001 is gastrointestinal. In general, decreased food consumption and corresponding decreases in body weight were the first signs of CMX001-related GI toxicity. In rats given a single 100 mg/kg dose, these signs of toxicity appeared two to three days after administration followed by dehydration, anorexia and nonformed/liquid/absent feces beginning six to eight days after administration. Histopathology changes in the GI were present as early as 24 hours after a 100 mg/kg dose, most severe at six to seven days post-dose, and fully resolved by 14 days post-dose. The histological findings were described as enteritis or enteropathy generally centered in the ileum or jejunum. In monkeys, gastropathy was also observed. Similar findings were observed at lower doses in repeat dose studies with daily administration for up to 14 days; however, no GI findings were observed in rats and monkeys administered CMX001 twice weekly for up to 13 weeks at doses of up to 15 mg/kg suggesting an extended dosing interval allows the GI time to recover between administrations.

CMX001 produced other toxicities that have previously been reported for cidofovir. CMX001 was positive in the chromosomal aberrations assay but negative in the Ames and *in vivo* micronucleus tests. Like cidofovir, there was a dose-related incidence of mammary adenocarcinomas in rats administered CMX001 however, no tumors were observed in a similar study in monkeys and no excess risk of carcinogenicity has been reported in humans administered cidofovir. Also like cidofovir, CMX001 had effects on reproduction and development including decreased fertility. Embryotoxicity, malformations (skeletal and visceral), and developmental effects including decreased body weight gain and delayed sexual maturation were observed at doses that caused maternal toxicity. In general, doses that did not produce these effects were identified and the dosing regimen required to produce the effects was either more frequent or longer than is anticipated for treatment of smallpox.

Although CMX001 exposures (AUC) in humans were higher than exposures in animals at comparable doses, the toxicities observed in animals correlated with dose rather than with exposure (AUC) and are expected to be predictive of potential human toxicities despite differences in exposure. For example, no effect doses as well as doses that caused severe toxicity were the same in mice and monkeys even though exposures at a given dose were about 10-fold lower in monkeys compared with mice. To date, no dose-limiting toxicities have been observed in more than 300 patients administered CMX001 at doses that bracket the anticipated dose for smallpox given for durations of up to six months which exceeds the anticipated treatment course for smallpox. Exposures in humans were higher than those observed in animals given similar doses, however, the doses were below those that caused dose-limiting toxicity in animals and the correlation with dose rather than exposure appears to be confirmed by the absence of any dose-limiting toxicities in humans administered CMX001 to date.

The most significant point of distinction between the toxicity of CMX001 and that of CDV, is nephrotoxicity, the dose-limiting toxicity of Vistide®. The clinical utility of cidofovir is limited by a risk of acute nephrotoxicity necessitating in-hospital administration by intravenous infusion preceded by hydration and renal protection using probenecid. Numerous endpoints were incorporated in the nonclinical development of CMX001 to monitor for potential effects on the kidney, including changes in serum chemistry (creatinine and BUN) and urinalysis values, changes in kidney weight, gross changes which included microscopic examination of the fresh cut (mid-line transverse section) surface of the kidney, and microscopic examination of fixed, stained sections by DACVP-certified veterinary pathologists. As judged by the absence of CMX001-related changes in any of these parameters, there was no evidence of cidofovir-like nephrotoxicity in any nonclinical study of CMX001. Hence, the dose-limiting toxicity of CMX001 appears to be gastrointestinal. In contrast to the nephrotoxicity produced by cidofovir, CMX001-related GI toxicity is easily monitored and rapidly reversible.

### 5.1. Species Differences and Similarities in CMX001 Pharmacokinetics and Metabolism

The pharmacokinetics of CMX001 has been evaluated in single and multiple dose studies in mice, rats, rabbits and cynomolgus monkeys [47,48]. CMX001 was readily absorbed in all species after oral administration, with lower systemic plasma concentrations observed in monkeys relative to other species including human (Table 4). The dose normalized exposure to CMX001 in humans (DN AUC_0–∞_) is more than 150-fold higher than that observed in monkeys. This difference is attributed, in part, to a higher rate of oxidative hepatic metabolism of CMX001 in monkeys.

The primary mode of elimination of CMX001 is via metabolism, based on lack of urinary elimination of CMX001 in animals and humans, as well as reasonably close predictions of *in vivo* clearance based on *in vitro* hepatocyte studies. After i.v. or oral administration of ^14^C-CMX001 to monkeys or mice, less than 0.1% of the dose is excreted as unchanged parent drug in urine. After oral administration of CMX001 to humans, CMX001 was below detection in most urine samples. After *in vitro* incubation with cryopreserved suspended hepatocytes, ^14^C-CMX001 was degraded more rapidly when incubated with cynomolgus or rhesus monkey hepatocytes than when incubated with rabbit or human hepatocyte incubations. Using intrinsic clearance obtained from this study to predict *in vivo* clearance based on the well-stirred model of hepatic clearance [49], a reasonably close prediction of cynomolgus monkey clearance is obtained (actual mean clearance values 22 and 25 mL/min/kg after i.v. administration of 1 and 4 mg/kg CMX001 to cynomolgus monkeys, respectively, compared to predicted 19 mL/min/kg), further supporting that metabolism by the liver is a major route of elimination for CMX001 [50]. The predicted *versus* actual clearance values for rabbits were 11 *versus* 16 mL/min/kg (i.v. administration of 4 mg/kg) and for humans were 6 *versus* 13 mL/min/kg (oral administration of 2 mg/kg).

The qualitative *in vitro* and *in vivo* metabolite profile was similar across species, though the quantities of metabolites differed between species. After incubation of ^14^C-CMX001 with mouse, rat, rabbit, monkey and human hepatocytes, the major metabolites determined by HPLC-MS-MS and radiochemical detection included CDV, CMX103 (3-(4-butanoic acid)propyl hydrogen ((S)-1-(4-amino-2-oxopyrimidin-1(2H)-yl)-3-hydroxypropan-2-yloxy)methylphosphonate), CMX064 (3-hydroxypropyl hydrogen ((S)-1-(4-amino-2-oxopyrimidin-1(2H)-yl)-3-hydroxypropan-2-yloxy)methylphosphonate); CMX108 (monohydroxylation on hexadecyl alkyl chain); and CMX108 glucuronide [50]. After administration of ^14^C-CMX001 to cynomolgus monkeys, the major metabolite in plasma and excreta was identified as CMX064; CDV, CMX103 and four additional oxidative metabolites (lipid chain-shortened carboxylic acids) were also detected in plasma and excreta by HPLC-MS-MS. After administration of ^14^C-CMX001 to mice, the major metabolite in plasma and excreta was CMX103, with lesser amounts of CDV and CMX064 also present. The concentrations of six metabolites (previously identified in animal ADME studies) were quantified in plasma samples following oral administration of CMX001 to humans (Clinical Study CMX001-103). The three major human metabolites were CMX103 (130% of CMX001 AUC), CDV (90% of CMX001 AUC) and CMX064 (50% of CMX001 AUC). The enzymes responsible for metabolism of CMX001 have not been fully elucidated. However, upon incubation of ^14^C-CMX001 with human hepatocytes in the presence of the cytochrome P450 (CYP) inhibitor, 1-aminobenztriazole, the amount of CMX103 formed was reduced by approximately 50%, suggesting that the formation of CMX103 was partially mediated by CYP enzymes [51].

### 5.2. OAT-mediated Secretion and Kidney Distribution

**Figure 3:**
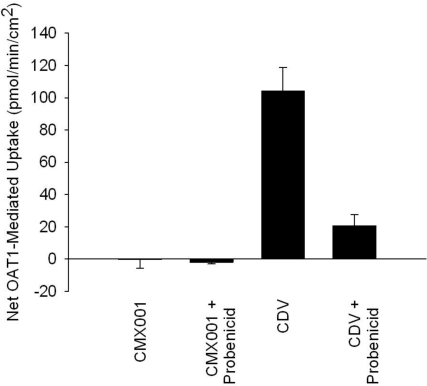
Uptake of CMX001 and CDV in OAT1-expressing cells *in vitro.*

CDV is actively secreted from blood into kidney proximal tubule cells by the organic anion transporters (OATs), leading to high concentrations in kidney proximal tubules and CDV-induced nephrotoxicity [52]. As shown in Figure 3 and in contrast to CDV, CMX001 uptake is not enhanced *in vitro* in cells expressing OAT1 compared to control cells that do not express this transporter (net uptake is approximately zero, after incubation with OAT1-expressing cells and non-OAT1-expressing control cells at a concentration of 5 μM of test drug for 5 min), nor is uptake reduced by the OAT inhibitor probenecid. These results indicate that CMX001 is not a substrate for OATs [53].

The distribution of radioactivity to the kidney seen in mice following administration of CMX001 is supportive of these *in vitro* findings. Peak concentrations of radioactivity in kidney cortex, the tissue which contains renal proximal tubules, was more than 30-fold lower after oral administration of 5 mg/kg ^14^C-CMX001 compared to that observed after i.v. administration of an equivalent dose of ^14^C-CDV. In addition, the tissue:plasma ratio in kidney cortex ranged from 4:1 at the time of peak concentration (4 h), and increased to 25:1 at 48 h after CMX001 administration compared to much higher ratios following i.v. cidofovir (21:1 at the time of peak concentration (0.5 h), with an increase to 400:1, 4 h after cidofovir administration). Thus, CMX001 has a low potential to cause CDV-induced nephrotoxicity because it is not itself a substrate for OATs [54].

## 6. Clinical Experience with CMX001

CMX001 is currently in clinical development for the prophylaxis and treatment of multiple dsDNA viral infections including: (1) the treatment of smallpox under the “Animal Rule” using animal efficacy data and human safety data, (2) prophylaxis/preemption of cytomegalovirus disease in human stem cell transplant (HSCT) recipients, and (3) preemption treatment of adenovirus disease in pediatric HSCT recipients. CMV and AdV are common, typically mild and self-limited, infections in the healthy host, but cause life- and graft-threatening disease in transplant patients as well as severe opportunistic infections in other immunocompromised individuals.

The demonstration of safety and efficacy of CMX001 in humans with these life-threatening dsDNA virus infections can provide critical supportive data for the registration package for the treatment of smallpox under the Animal Rule. In draft guidance for industry entitled “Smallpox (Variola) Infection: Developing Drugs for the Treatment or Prevention” published in November of 2007, FDA states that “because smallpox is no longer a naturally occurring disease, data from studies of a candidate in other human illnesses might play a more important role than usual in the evaluation of drug safety.” In this same guidance FDA also states that human data are particularly compelling if they involve the study of “viral infections related to variola.” Certainly in the case of CMX001, pharmacokinetic and pharmacodynamic parameters for doses that demonstrate clinical efficacy for the treatment of dsDNA viral infections can be compared directly to pharmacokinetic and pharmacodynamic parameters necessary for the prevention of mortality in animal models of human smallpox (*i.e.*, animals infected with dsDNA viruses in the family *Orthopoxviridae*). This comparison will validate that drug exposures necessary to treat smallpox can be achieved in humans and that these doses are efficacious in humans as evidenced by activity against similar dsDNA virus infections.

CMX001 has been administered to healthy human volunteers in two completed Phase 1 clinical trials (designated CMX001-102 and CMX001-103). Two clinical trials are ongoing: a Phase 1b/2a trial (CMX001-104) in renal and hematopoietic stem cell transplants (HSCT) patients with BK virus viruria, and a Phase 2 trial (CMX001-201) in HSCT recipients to evaluate CMX001 for the prophylaxis and/or pre-emptive treatment of CMV infection in this population. In addition over 105 patients have been treated for various dsDNA virus infections under Emergency INDs or the ex-US equivalent.

### 6.1. CMX001-102

CMX001–102 was a Phase 1, dose-escalation, pharmacokinetic (PK), first time in humans (FTIH) study of the safety and tolerability of CMX001 in healthy human volunteers. A solution formulation of CMX001 was administered as a weight-based dose to volunteers in a total of nine single dose cohorts (.025, .05, 0.1, 0.2, 0.4, 0.6, 1, 1.5 and 2 mg/kg) and five multiple dose cohorts (0.1, 0.2, 0.4, 0.6 and 1.0 mg/kg). Subjects in the multiple dose cohorts received a total of three doses of CMX001, one dose every six days. Each cohort enrolled six subjects randomized 2:1 (active: placebo). Following the completion of each cohort, all safety data and subsequent dose escalations were reviewed and approved by a Data Safety Monitoring Board (DSMB) and the FDA. Safety analysis included clinical and laboratory assessments as well as wireless capsule endoscopy (WCE) which was conducted before and after dosing to look for macroscopic evidence of GI toxicity.

There were no severe adverse events (SAEs), no adverse events (AEs) that prevented dose escalation, and no evidence of GI toxicity. A total of 30 treatment-emergent adverse events were reported by 16 subjects (30% of subjects who received at least one dose of CMX001 or placebo). Among the 36 subjects who received CMX001, nine (25%) subjects reported at least one adverse event and among the 18 subjects who received placebo, seven (39%) subjects reported at least one adverse event. No dose-related trend in the incidence or severity of AEs was observed.

After a single dose under fasting conditions, CMX001 was readily absorbed with the time to maximum plasma concentration (T_max_) ranging from two to three hours. Maximum plasma concentration (C_max_) and systemic exposure (AUC) increased approximately in proportion to dose over the range of 0.025 to 2.0 mg/kg. The half-life of elimination (t_1/2 elim_) increased with increasing dose, ranging from 6.15 hours at 0.025 mg/kg to 32.7 hours at 1.5 mg/kg, presumably due to better definition of the elimination phase at higher doses. CMX001 is not eliminated in urine; however, CDV, a metabolite of CMX001 is eliminated in urine. No changes in PK parameters were observed after repeat administration.

### 6.2. CMX001-103

CMX001-103 was a Phase 1 comparative bioavailability study of CMX001 solution *versus* tablets, plus a comparison of PK parameters for CMX001 and CDV in subjects administered CMX001 after fasting overnight *versus* having eaten a high fat meal within 30 minutes of dosing. CMX001 was administered as a fixed dose to 24 volunteers in three single doses (40 mg solution, fasted; 40 mg tablet following a high fat breakfast; and 40 mg tablet fasted). Each dose was separated by a 14-day washout period.

CMX001 was generally safe and well tolerated by healthy volunteers. The most frequently reported adverse events were headache (17%), increased blood CPK (17%), increased ALT (13%), nausea (8%), and oropharyngeal pain (8%). This study showed that CMX001 administered as a tablet in the fasted state produces similar C_max_ and AUC values to those observed after CMX001 administration as solution in the fasted state, and that CMX001 administered as tablet in the fed state as compared with the fasted state demonstrates a significant food effect as evidenced by decreased C_max_, AUC_∞_ and AUC_last_ values. Peak plasma concentrations (C_max_) and systemic exposure (AUC_0–∞_) following administration in the fed state were reduced approximately 48% and 28%, respectively, compared with C_max_ and AUC_0–∞_ following administration to fasted subjects. In addition, the median time to peak plasma concentrations in fed subjects was approximately twice that for fasted subjects. Changing the dose formulation from solution to tablet may reduce exposure to CMX001 by a maximum of approximately 13%.

### 6.3. CMX001-104

CMX001-104, a study of the safety, tolerability, and preliminary antiviral activity of CMX001 in renal transplant and HSCT recipients with BK viruria, is nearing completion. A total of twenty-eight subjects were enrolled into this study. All received fixed doses of 10, 20, or 40 mg of CMX001 or placebo once or twice weekly for up to 28 days. No serious adverse events attributable to study drug (*i.e.*, CMX001 or placebo) have been reported.

### 6.4. CMX001-201

CMX001-201, a multicenter, randomized, double-blind, placebo-controlled, dose-escalation study of the safety, tolerability, and ability of CMX001 to prevent or control post-transplant CMV infection in CMV seropositive HSCT recipients is underway. Up to 6 dose-escalation cohorts of 32 subjects per cohort are planned. An expansion phase of this study is also planned to enroll up to 200 patients at a dose assessed during the dose-escalation phase and selected based on preliminary safety and efficacy. The expansion phase will increase enrollment at the selected dose to obtain the statistical power needed to show a difference in efficacy between CMX001 and placebo in the prevention of CMV. Forty subjects were enrolled in cohort 1 and 39 subjects were enrolled in cohort 2. Subjects were randomized 3:1 to receive once weekly doses of 40 mg (cohort 1) or 100 mg (cohort 2) of CMX001 or matching placebo. Treatment with study drug was continued for up to 90 days (or 13 weeks) post-transplant. Enrollment into cohort 3, at a once weekly dose of 200 mg CMX001 or placebo is ongoing.

### 6.5. Emergency IND Experience

Additional clinical experience with CMX001 has been gained through the emergency treatment of various dsDNA virus infections under Emergency IND (EIND) or ex-US equivalent [55–57]. More than 105 patients have received CMX001 through emergency single patient protocols. Patients from one month to 69 years of age have been treated, with the longest treatment duration in excess of six months. Results of the EIND experience have been reported for the use of CMX001 to treat recurrent CMV (a case series involving three transplant patients who had failed valganciclovir therapy), AdV infection (a case series of thirteen immunocompromised patients with a median age of 12 years, range 0.75 to 66, with disseminated AdV infection) and progressive vaccinia (a single case with complications from smallpox vaccination). For example, AdV viremia declined by almost 1000-fold (2.9 log_10_) in patients who received eight weeks of CMX001 therapy [55].

In an analysis of pharmacokinetic data from 49 patients treated under EINDs with CMX001, higher clearance resulting in lower systemic exposure (AUC_0–∞_) was observed in pediatric (age ≤ 18 years) patients relative to adults at doses of 2, 3 and 4 mg/kg. At a given dose, pediatric patients typically had 30% to 50% of the average adult exposure. In both pediatric and adult patients, C_max_ and AUC_0–∞_ generally increased greater than in proportion to dose. In a second analysis conducted separately for both pediatric and adult patients, systemic exposure to CMX001 was comparable between patients with normal renal function and those with moderate to severe renal impairment, in both the pediatric and adult populations, indicating that dose adjustment is not needed in patients with renal impairment. In this analysis, it was found that CMX001 exposure is not affected by hemodialysis [48].

### 6.6. Additional Studies in Planning Stage

Two additional clinical studies are under development. A randomized, double-blind, placebo-controlled study evaluating the safety and efficacy of pre-emptive treatment with CMX001 in children for the prevention of AdV disease following HSCT is planned. Also, a multicenter, open-label study of CMX001 treatment of serious diseases or conditions caused by dsDNA viruses is in the initial stages of enrollment. In the open-label study, up to 200 patients without viable alternatives, including other studies of CMX001, will be enrolled for the treatment of diseases caused by poxviruses (vaccinia virus and molluscum contagiosum), adenoviruses, herpesviruses (CMV, HSV, EBV, VZV, HHV6), polyomaviruses (BKV or JCV), and HPV. Treatment with CMX001 in this protocol may be continued twice weekly for up to six months, as needed, based on the clinical disease state of the patient.

## 7. Conclusions

CMX001 has demonstrated *in vitro* and *in vivo* efficacy against orthopoxvirus infections. In the rabbitpox model it significantly improved survival even when administered after lesions were detected. Although the antiviral activity of CMX001 is not evaluable in monkeys due to species specific metabolism/anabolism issues which result in low intracellular levels of the active antiviral, CDV is an effective surrogate for CMX001 and scaling animal results to humans via CDV-PP should allow development to proceed using the variola model in monkeys combined with proof of efficacy against similar dsDNA virus infections in humans. Current data suggest the 2 mg/kg dose of CMX001 which has been used to treat dsDNA virus infections in patients should produce CDV-PP levels above those attained in rabbits dosed with levels of CMX001 which effectively prevented mortality after appearance of lesions. The reductions in adenovirus viremia reported in EIND patients following treatment with CMX001 provide further support for potential human efficacy from a non-orthopox dsDNA virus model that has important similarities to VARV, including disseminated, lytic infection of epithelial cells. The emerging efficacy, safety, pharmacokinetic and resistance profiles of CMX001 make it a promising antiviral for use in the treatment of smallpox.

## Figures and Tables

**Table 1:** The *in vitro* activity of CMX001 and CDV against viruses in all five families of dsDNA viruses known to cause human morbidity and mortality.

**Viral Class**	**Virus (ref)**	**CMX001 EC_50_ (μM)**	**CDV EC_50_ (μM)**	**Enhanced Activity (EC_50_ CDV/EC_50_ CMX001)**
Adenovirus	AdV 5 [17]	0.02	1.3	65
Herpesvirus	HSV 1 [18]	0.06	15	250
	HHV 6 [18]	0.004	0.2	50
	CMV [18]	0.0009	0.38	422
	VZV [18]	0.0004	0.5	1250
	EBV [18]	0.04	>170	>4250
Papillomavirus	HPV 11 [19]	17	200	12
Polyomavirus	BKV [20]	0.13	115.1	885
	JCV [21]	0.045	nd	n/a
Orthopoxvirus	VARV [22]	0.1	27.3	271
	VACV [23]	0.8	46	57
	ECTV [24]	0.5	12	24
	RPXV [25]	0.5	39	78
	MPXV [22]	0.07	4.6	65

nd: not determined; n/a: not applicable; Abbreviations for viruses: AdV, adenovirus; HSV, herpes simplex virus; HHV, human herpes virus; CMV, human cytomegalovirus; VZV, varicella-zoster virus; EBV, Epstein-Barr virus; HPV, human papillomavirus; BKV, BK virus; JCV, JC virus; VARV, variola virus; VACV, vaccinia virus; ECTV, ectromelia virus; RPXV, rabbitpox virus; MPXV, monkeypox virus; cowpox virus, CPXV.

**Table 2:** Mutations in the polymerase gene of VACV associated with CDV resistance. Amino acid numbering is according to VACV strain WR DNA polymerase (GenBank accession No. P06856).

**Mutation(s)**	**CDV Fold Resistance**	**Author**	**Virulence in Mice**
ΔK174	3 to 4	Becker [29]	Reduced
A314T	5 to 7	Andrei [28]	Reduced
A314V	7	Becker [29]	Reduced
A684V	3 to 9	Andrei [28]; Gammon [30]	Reduced
S851Y	2 to 3	Gammon [30]	Reduced
H296Y/S338F	12	Kornbluth [27]	nd
A314T/A684V	11 to 15	Andrei [28]	Reduced
A314T/T688A	11 to 17	Andrei [28]	Essentially non-virulent
A684V/S851Y	7 to 16	Gammon [30]	Reduced
ΔK174/M671I	4 to 5	Becker [29]	Reduced
A314T/A684V/ Y232H	25	Andrei [28]	nd
H296Y/A314V/H319N/S338F/ R604S	11 to 14	Kornbluth [27]	Reduced

nd: not done.

**Table 3:** Efficacy of CMX001 in protecting rabbits from mortality following a lethal intradermal inoculation with rabbitpox virus.

**Treatment Regimen**	**Total CMX001 Dose**	**CMX001 Survivors**	**Placebo Survivors**	P Value*
3 doses of 20 mg/kg CMX001 (1 dose every other day starting at onset of lesions)	60 mg/kg	11/12	2/12	0.0006
2 doses of 20 mg/kg CMX001 (1 dose every other day starting at onset of lesions)	40 mg/kg	8/12	1/12	0.0049
1 dose of 20 mg/kg CMX001 at onset of lesions:	20 mg/kg	7/12	1/12	0.0272

* 2-sided Fisher’s exact test.

**Table 4:** Plasma C_max_ and AUC of CMX001 and CDV after a single oral administration of CMX001 to animals and humans.

**Species**	**Dose (mg/kg)**	**CMX001**			**CDV**		
		**C_max_ (ng/mL)**	**AUC_0–∞_ (h·ng/mL)**	**DN^1^ AUC_0–∞_**	**C_max_ (ng/mL)**	**AUC_0–∞_ (h·ng/mL)**	**DN^1^ AUC_0–∞_**
Monkey	4	13	32	8	14	197	49
Rat	4	14	141	35	9	280	70
Mouse	10	69	403	40	11	175^2^	17^2^
Rabbit	4	30	179	45	13	254^2^	66^2^
Human	2	350	2650	1325	31	1740	870

1 dose normalized;

2 AUC_0–24h_.
